# Exploring the Effect of Jiawei Buguzhi Pills on TGF-*β*-Smad Pathway in Postmenopausal Osteoporosis Based on Integrated Pharmacological Strategy

**DOI:** 10.1155/2021/5556653

**Published:** 2021-10-31

**Authors:** Xiao Yuan, Yonghe Wu, Kailin Yang, Huiping Liu, Guomin Zhang

**Affiliations:** Hunan University of Chinese Medicine, Changsha, Hunan Province, China

## Abstract

**Objective:**

To explore the effect of Jiawei Buguzhi Pills (JWBGZP) on the TGF-*β*-Smad pathway in postmenopausal osteoporosis (PMO) based on integrated pharmacological strategy.

**Method:**

The ETCM database was used to collect JWBGZP. GeneCards and OMIM databases were utilized to obtain PMO-related genes. Cytoscape was used for network construction and analysis, and DAVID was used for GO and KEGG enrichment analysis of key targets. Animal experiments and cell experiments were conducted to further explore the mechanism. The bone mass density was detected by dual-energy X-ray bone densitometer. The TGF-*β*1 and Smad4 mRNA in bone tissue were detected by RT-qPCR. The TGF-*β*1 and Smad4 protein in bone tissue were detected by the western blot. The TGF-*β*1 and Smad4 protein in osteoblasts were determined by immunohistochemistry.

**Result:**

A total of 721 JWBGZP potential targets and 385 PMO-related genes were obtained. The enrichment analysis showed that JWBGZP may regulate the TGF-beta signaling pathway, oxidation-reduction process, aging, response to hypoxia, response to ethanol, negative regulation of cell proliferation, PI3K-Akt, HIF-1, and other signaling pathways. The animal experiments showed that compared with the model group, the femoral bone mineral density and lumbar bone mineral density of the JWBGZP group increased (*P* < 0.05); the expression levels of TGF-*β*1 and Smad mRNA and proteins in the JWBGZP group were significantly higher (*P* < 0.05). The cell experiment results showed a large number of osteoblast stained blue-purple and orange-red calcified nodules. The expression levels of TGF-*β*1 and Smad proteins in the JWBGZP group were significantly higher than those in the blank control group and the sham operation group, and the protein expression levels in the model group were the lowest (*P* < 0.05).

**Conclusion:**

JWBGZP may be involved in PI3K-Akt, HIF-1, estrogen, prolactin, and other signaling pathways and regulate MAPK1, AKT1, PIK3CA, JAK2, and other gene targets, regulate bone metabolism, and thereby treat PMO.

## 1. Introduction

Postmenopausal osteoporosis (PMO) is a metabolic bone disease that usually occurs 3–4 years after menopause. The sharp drop in estrogen levels after menopause leads to bone resorption greater than bone formation, resulting in bone loss. The patient usually has no obvious symptoms, but due to the loss of bone mass, it is prone to fragility fractures [1, 2]. Clinically, PMO often presents lower back pain or leg pain, which is chronic and persistent and can be aggravated by coughing and bending over [3]. In severe cases, there may be a series of symptoms such as hunchback or shortening of height due to vertebral compression fractures. Epidemiological studies have shown that China has over 210 million people over 60 years old, and there are approximately 140 million people aged 65 and over [4]. In China, among the elderly, 60–69 years old, osteoporosis in women accounts for about 50% to 70%, and about 30% in men [5]. By 2050, medical expenses for fractures in Europe will reach approximately 7.5 billion euros [6]. A series of complications such as bone deformation and fracture caused by PMO not only affect the physical and mental health and quality of life of postmenopausal women, but also cause a heavy family and social burden [6]. In postmenopausal women, due to the decrease in estrogen secretion, oxidative stress response occurs in the body. The large amount of active oxygen not only inhibits bone formation, but also promotes bone resorption, which eventually causes bone loss [7]. At present, the treatment and prevention drugs for PMO mainly include calcium, vitamin D, antibone resorption drugs, and bone active drugs [6, 8–10]. Although they play an important role in the treatment of osteoporosis, there are also problems such as drug side effects and insufficient patient compliance. On the other hand, Chinese medicine (TCM) is based on the whole and seeks the root of the disease. TCM is gradually playing more roles in the treatment of osteoporosis [11, 12].

Buguzhi pills from “Compendium of Materia Medica” is a classic prescription for strengthening the kidney and strengthening the bones. Current studies have shown that *Psoralea* corylifolia Linn has an estrogen-like effect, maintains bone density, inhibits bone resorption, and promotes bone formation in patients with PMO, and may improve the body's bone metabolism balance [13–16]. Jiawei Buguzhi pills (JWBGZP) are modified from Buguzhi Pills, in order to further explore the potential mechanism of JWBGZP in the treatment of PMO. Network pharmacology is based on the theory of system biology and the network analysis of biological systems [17, 18]. Network pharmacology emphasizes the multichannel regulation of signal pathways because it is consistent with the characteristics of the overall concept of Chinese medicine (TCM). It provides an effective research approach for predicting the core targets and pathways of drug treatment of diseases and is especially suitable for the research of complex systems such as TCM or chronic diseases [19,20]. At present, through the application of system biology strategies in the analysis of TCM compound prescriptions, not only has the TCM drug compatibility law been verified at the system level, but it has also played an important role in finding potential active compounds [21, 22]. This study uses network pharmacology methods to explore the potential effective components and targets of JWBGZP in the treatment of PMO and analyze the key targets of gene ontology functions and signal pathways to provide references for future anti-PMO treatments and in-depth research.

## 2. Materials and Methods

### 2.1. JWBGZP Potential Targets and PMO Gene Collection

JWBGZP consists of *Fructus Psoraleae* (Bu Gu Zhi) 15 g, *Epimedii Folium* (Yin Yang Huo) 15 g, *Lycii Fructus* (Gou Qi) 10 g, *Cyathulae Radix* (Chuan Niu Xi) 10 g, *Fructus Ligustri Lucidi* (Nv Zhen Zi) 10 g, *Rhizoma Cibotii* (Gou Ji) 10 g, and *Rhizoma Drynariae* (Gu Sui Bu) 10 g. The herbs of JWBGZP were used as a keyword to search in ETCM (http://www.tcmip.cn/ETCM/index.php/Home/Index/) [23] (Table S1). “Postmenopausal osteoporosis” was used as a search term to search for PMO-related genes in the GeneCards [24] and OMIM databases [25], respectively. The search results of the two data are summarized, and duplicate genes are removed, which is the target of the PMO disease (Table S2).

### 2.2. Network Construction and Analysis

JWBGZP potential targets and PMO genes were input into STRING database (https://string-db.Org/), organization is set to “*Homo sapiens*” to obtain protein-protein interaction (PPI) data. The PPI data is imported into Cytoscape to construct the network and perform topology analysis. JWBGZP potential targets and PMO genes were imported into the DAVID 6.8 database (https://david.ncifcrf.gov/) [26], and the gene ontology (GO) function enrichment analysis and KEGG pathway enrichment analysis were performed, respectively. Among them, GO analysis includes three contents: biological process (BP), molecular function (MF), and cellular component (CC).

### 2.3. Experimental Materials

#### 2.3.1. Experimental Animals

1. Animal for animal experiments: 75 SD female rats, body weight (200 ± 10) g, were provided by Guangdong Medical Laboratory Animal Center (license number: SCXK (Guangdong) 2018-0002).
2. Animal for cell experiments: 60 SD female rats, body weight (200 ± 10) g, were provided by Guangdong Medical Laboratory Animal Center (license number: SCXK (Guangdong) 2018-0002). 10 SD rats born within 72 hours were provided by the Experimental Animal Center of Hunan University of Chinese Medicine (Certificate Number: SCXK (Xiang) 2019-0004). The experiment was approved by the Animal Ethics Committee of Hunan University of Chinese Medicine (Ethics approval number: LLBH-202103050004).

#### 2.3.2. Instruments and Reagents

JWBGZP extract (prepared by the Department of Natural Medicine, Central South University) is a mixture of oil phase and water phase. Psoralen (batch number: 110739-201817, purity: 99.7%) and isopsoralen (batch number: 110738-201814, purity: 99.9%) were purchased from China Institute for Food and Drug Control.

DEME low-sugar solution, penicillin-streptomycin mixed solution, and type II collagenase (Shanghai Lifei Biotechnology Company) were used. The serum E2 kit was purchased from Wuhan Boster Biology Co. Ltd. Fetal bovine serum was purchased from Zhejiang Tianhang Biological Technology Co. Ltd; alkaline phosphatase staining solution and Alizarin red staining solution were purchased from Progema Biotechnology Company. TGF-*β*1 ELISA kit was purchased from Blue Gene Biotech Co. (lot number: EK3812/2); TGF-*β*1-anti (Santa Cruz Biotechnology, lot number: sc-130348). HRP-labeled goat anti-rabbit IG was purchased from Abcam, UK). RIPA protein lysate and protease inhibitor were purchased from Beyotime Biotechnology (product number P0013B (P1030)). Goat anti-rabbit IgG was purchased from Zhongshan Jinqiao (batch number: ZB-2301). *β*-Actin antibody (anti-beta actin antibody [mAbcam 8226]-loading control (ab8226)) was used. PCR primers were provided by Shanghai Bioengineering Co. Ltd. Animal tissue total RNA extraction kit (TianGEN, lot number: Q5301) and 2 × SYBR Premix ExTaqTM (TianGEN, lot number: Q6220) were used. 1260 type high-performance liquid chromatography (HPLC), including G1329B autosampler, G1315D diode array detector, G1311C quaternary gradient elution pump, G1316A column thermostat, and 1260 chromatography workstation (Agilent, USA) was used; low-temperature centrifuge, 37°C thermostat, DNP-9162 electric heating thermostat incubator, and LEICA DM LB2 binocular microscope were used; dual energy X-ray bone densitometer (Unigamma X-ray Plus), Motic B microscopic camera system, and MIAS medical image analysis system were also used.

### 2.4. JWBGZP Quality Control by HPLC

#### 2.4.1. Solution Preparation

Reference solution: an appropriate amount of psoralen and isopsoralen reference substance was accurately weighed and placed in the same 50 mL measuring flask, to which methanol-0.1% acetic acid solution (1 : 1, V/V) was added to dissolve and dilute to volume. Then, 1 ml of it was taken and placed in a 10 ml measuring flask, and methanol-0.1% acetic acid solution (1 : 1, V/V) was added to make the volume constant. JWBGZP solution was prepared from the JWBGZP extract.

#### 2.4.2. HPLC Condition

**Figure 1:**
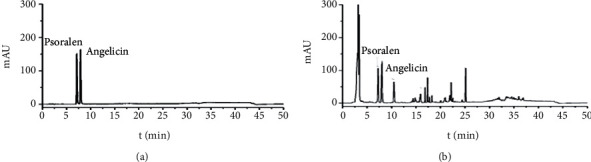
HPLC results: (a) reference solution; (b) JWBGZP solution.

HPLC conditions were as follows: column: Kinetex-C1 (250 mm × 4.6 mm, 5 um); mobile phase: methanol (A)-0.1% acetic acid solution (B), gradient elution 1; flow rate: 1.0 mL/min; detection wavelength: 248 nm; column temperature: 30°C; and injection volume: 20 uL. The results are shown in Figure 1.

### 2.5. Experimental Methods for Animal Experiments

#### 2.5.1. Animal Grouping, Modeling, and Intervention

The female rats were randomly divided into sham operation group, model group, JWBGZP low dose, medium dose, and high dose groups with 15 rats each. Modeling was carried out according to the literature method [3]. The rats were anesthetized with 2% sodium pentobarbital (0.2 mL/100 g), and the ovaries were harvested by making approximately 1 cm incisions on both sides of the lumbar spine. In the sham operation group, only the same amount of fat was cut, and the abdominal wall was sutured in layers and sterilized with ethanol. 800,000 U of penicillin was given daily within 3 days after treatment. After the operation, the rats received vaginal smears every day to observe the changes in the estrus cycle. Rats with estrous cycle disorders are considered to be successful in ovariectomy.

The bone density was measured by using the dual-energy X-ray absorption method, and the rats with bone density reduction were selected as the successful model of postmenopausal osteoporosis. 12 weeks after modeling, rats were given intragastric administration. The dosage is calculated based on the body surface area of humans and rats. The high dose of JWBGZP is equivalent to 13.2 g/kg of crude drug, the medium dose is equivalent to 6.6 g/kg of crude drug, and the low dose is equivalent to 3.3 g/kg of the crude drug. The administration volume was 1 mL/100g, and the model group and the sham operation group were given equal volumes of purified water. The administration lasted for 13 weeks.

#### 2.5.2. Serum E2 Level Detection

After 13 weeks of administration, rats in each group were anesthetized by intraperitoneal injection of 2% sodium pentobarbital (0.2 mL/100 g), blood was collected from the abdominal aorta, and serum was separated. Serum E2 was measured by using radioimmunoassay.

#### 2.5.3. Femur Tissue mRNA Expression Detected by RT-qPCR

The femoral tissue was ground in liquid nitrogen, and 1 ml TRlzol was added to extract total RNA from the tissue. NanoDrop 2000 spectrophotometer was used to detect the purity and concentration of total RNA; PrimeScriptMRT kit and gDNA Eraser were used to reverse transcribe RNA into cDNA. Amplification conditions were as follows: 95°C predenaturation for 5 min: 95°C denaturation for 15 s, 60°C extension for 30 s, repeat 40 cycles. Finally, the expression levels of TGF-*β*1, Smad4, and *β*-actin mRNA were calculated according to the 2-△△ method. The RT-PCR primer sequences are shown in Table 1.

#### 2.5.4. Femur Tissue Protein Expression Detected by Western Blot

50 mg of femoral bone tissue was extracted by RIPA protein lysate according to the manufacturer's instructions, and the protein was quantified using the BCA method. After the separation of the proteins by SDS-PAGE electrophoresis, the proteins were transferred to a polyvinylidene (PVDF) membrane and blocked with 5% skimmed milk powder for 60 min. Afterwards, the PVDF membrane was incubated with primary antibodies (TGF-*β*1 Smad4 and *β*-actin, diluted 1 : 1000) at 4°C overnight, and the secondary antibody (HRP-labeled goat anti-IG, diluted 1 : 10000) was incubated for 90 min. Finally, the ECL reagent was used for chemiluminescence development, and Image J was used to analyze the gray value.

### 2.6. Experimental Methods for Cell Experiments

#### 2.6.1. Animal Grouping and Modeling

The female rats were randomly divided into blank control group, sham operation group, model group, and JWBGZP group with 15 rats each. Modeling and model assessment were carried out according to Section 2.5.1.

#### 2.6.2. Extraction and Culture of Osteoblasts

Ten SD rats born within 72 hours were anesthetized and sacrificed by cervical dislocation. They were immersed in absolute ethanol for 20 s, and the skulls were cut off aseptically in PBS buffer and rinsed. Then, it was cut into 1 mm × 1 mm bone pieces and placed in 4 mL 0.25% trypsin for digestion at 37°C for 30 min. The bone fragments were transferred into a centrifuge tube containing 0.1% type I collagenase, digested in a constant temperature water bath at 37°C for 1 h, and centrifuged at 1,000 r/min for 10 min twice. Then, they were added into the culture medium in aliquots, placed in a volume fraction of 5% CO_2_, and incubated at 37°C. The medium was changed for the first time after 3 days, and the medium was changed every 3 days thereafter. Then, it was digested with 0.25% trypsin, the cells were passaged in a 1 : 1 manner, and the 3rd generation cells were used for subsequent experiments.

#### 2.6.3. Drug-Containing Serum Preparation

Intervention began in the 3rd week after modeling. The JWBGZP group was given 1.5 mL of JWBGZP every day, and the other groups were given 1.5 mL of normal saline for 12 consecutive weeks. The dose of the drug was corrected once a week according to body weight. 24 hours after the last administration, 2% sodium pentobarbital was injected intraperitoneally, and 8 mL of blood was taken from the main abdominal artery. The blood sample was placed at 4°C for 4 h, centrifuged at 3,000 r/min for 30 min, the supernatant was taken, and inactivated at 56°C for 30 min. The sera of the same group were mixed, filtered through a 0.22 *μ*L sterile filter and aliquoted, and stored at −20°C.

#### 2.6.4. Drug-Containing Serum Intervention

The third-generation osteoblasts were digested with 0.25% trypsin, and the cell suspension was inoculated into a 24-well culture plate at a final concentration of 2 × 10^4^/mL, divided into 4 × 4 wells. After 24 hours of cell attachment, 1 mL of their respective serum was added, and the medium was changed for 3 days during the culture. The culture supernatant was collected when the medium was changed for the first time and stored at −20°C for later use for immunohistochemical detection.

### 2.7. Statistical Analysis

The SPSS 20.0 software was used for statistical analysis, and the measurement data were expressed as “mean ± SD”. The comparison was performed by the *t*-test, and *P* < 0.05 was considered statistically significant.

## 3. Results and Discussion

### 3.1. JWBGZP Potential Targets and PMO-Related Genes

**Figure 2:**
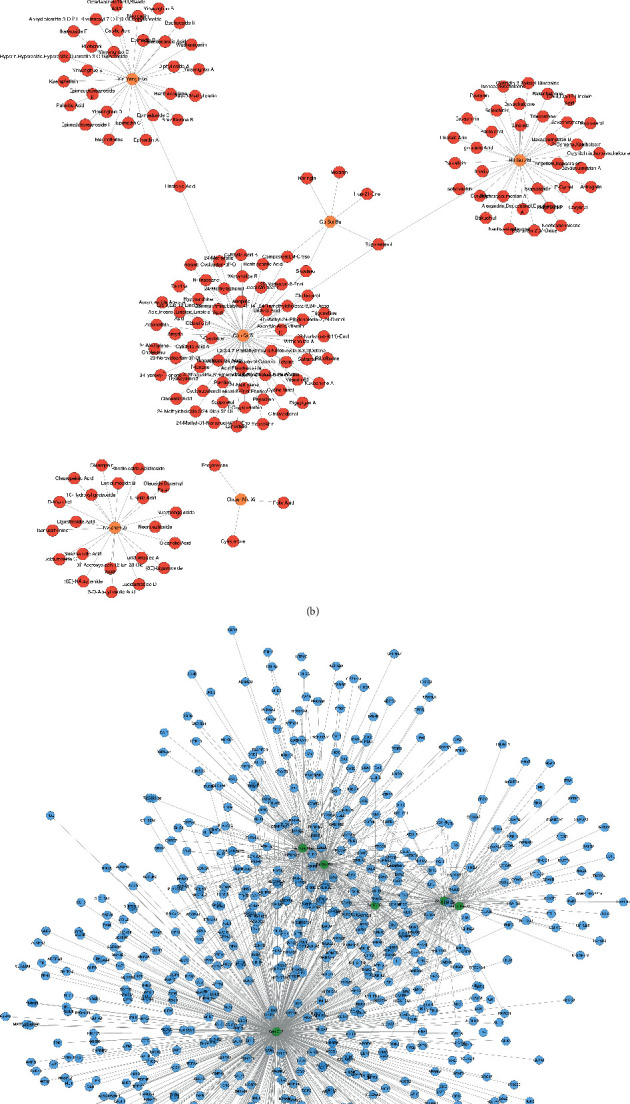
JWBGZP potential components and targets. (a) Herb-meridian tropism network; red circles stand for meridian tropism; orange diamonds stand for herbs. (b) Herb-component network; orange circle stands for herb; red circle stands for component. (c) Herb-potential target network; green circle stands for herb; blue circle stands for potential target.

**Figure 3:**
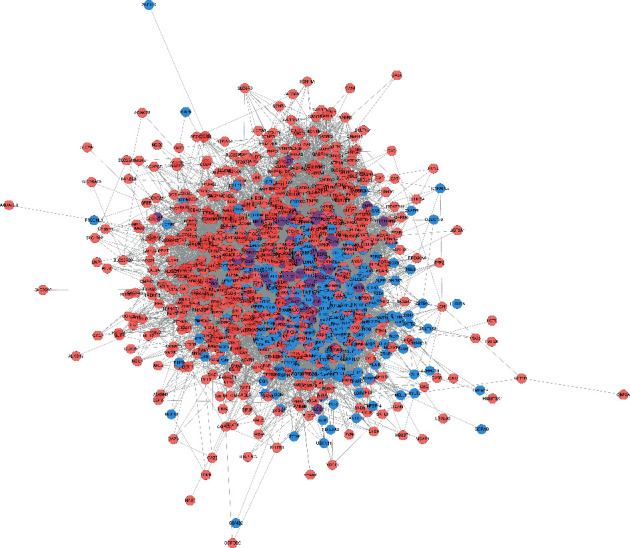
JWBGZP-PMO PPI network (pink circle stands for the JWBGZP target, blue circle stands for the PMO gene, and purple circle stands for the JWBGZP-PMO target).

Bu Gu Zhi is warm and pungent; it returns to the kidney meridian. Yin Yang Huo is warm, sweet, mildly bitter, and pungent; it returns to the liver meridian, triple burner meridian, and kidney meridian. Chuan Niu Xi is even, sweet, bitter, and sour; it returns to the kidney meridian and liver meridian. Gou Qi Zi is even and sweet; it returns to kidney meridian and liver meridian. Nv Zhen Zi is cool, sweet, and bitter; it returns to kidney meridian and liver meridian. Gu Sui Bu is warm and bitter; it returns to kidney meridian and liver meridian (Figure 2(a)). A total of 150 JWBGZP components, 721 JWBGZP potential targets, and 385 PMO-related genes were obtained. The JWBGZP herbs, components, and potential targets are shown in Figures 2(b) and 2(c). The JWBGZP potential target, PMO-related gene, and JWBGZP-PMO PPI data were input into Cytoscape 3.8.2 to construct the JWBGZP-PMO PPI network. This network consists of 610 JWBGZP target nodes, 285 PMO gene nodes, 55 JWBGZP-PMO target nodes, and 20658 edges (Figure 3). The top 30 targets were divided into 3 categories: (1) JWBGZP target set: GAPDH (350 edges), DECR1 (184 edges), CASP3 (179 edges), HRAS (178 edges), and CREB1 (167 edges); (2) PMO gene set: ALB (305 edges), IL6 (296 edges), MYC (226 edges), EGF (217 edges), STAT3 (203 edges), IGF1 (200 edges), JUN (199 edges), FOS (192 edges), CXCL8 (190 edges), CTNNB1 (187 edges), IL1B (181 edges), IL10 (177 edges), MMP9 (175 edges), PTEN (174 edges), CAT (173 edges), and LEP (166 edges); (3) JWBGZP-PMO target set: INS (355 edges), AKT1 (327 edges), TP53 (291 edges), VEGFA (256 edges), MAPK3 (254 edges), TNF (249 edges), SRC (213 edges), ESR1 (187 edges), and PTGS2 (170 edges).

### 3.2. Enrichment Analysis of JWBGZP-PMO PPI Networks

**Figure 4:**
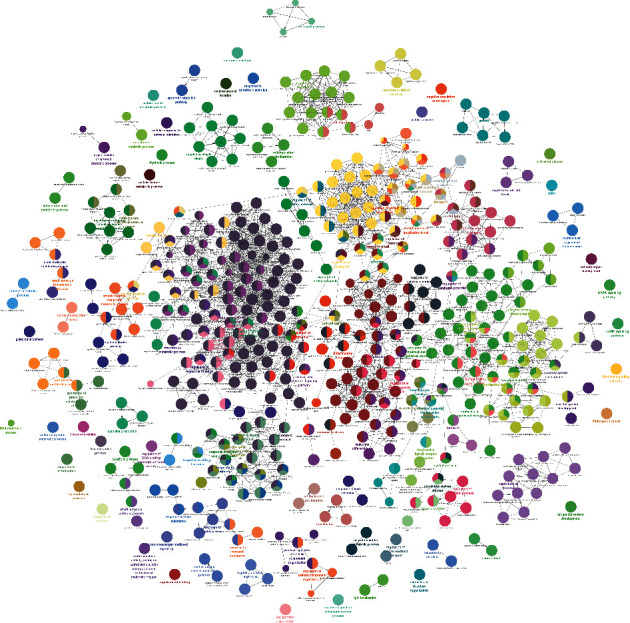
The results of enrichment analysis.

**Figure 5:**
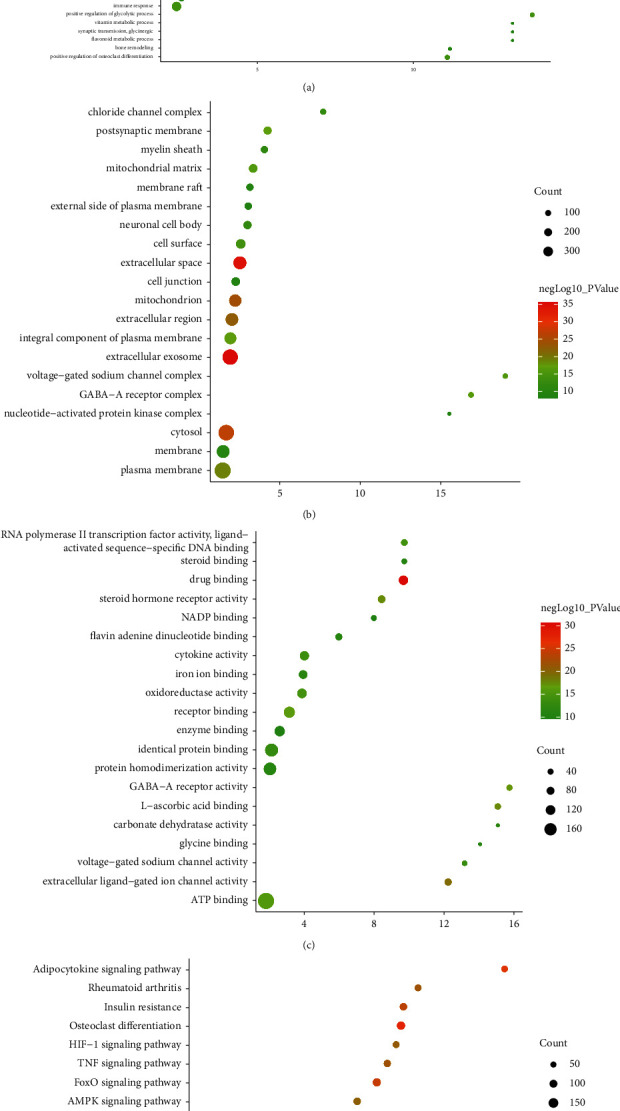
Bubble chart: (a) biological processes; (b) cell components; (c) molecular function; (d) signaling pathway. *X*-axis is fold enrichment.

**Figure 6:**
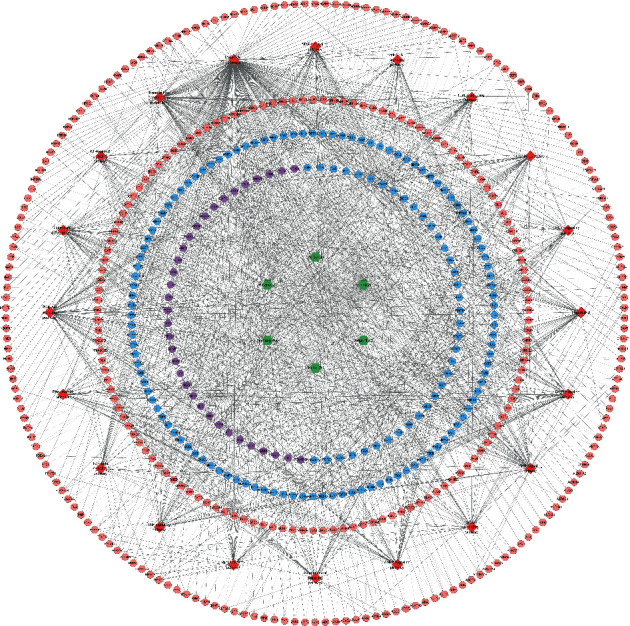
Herb-target-signaling pathway network (pink, blue, and purple circles stand for JWBGZP target, PMO gene, and JWBGZP-PMO target, respectively. Green hexagon stands for herbs. Red diamond stands for the signaling pathway).

**Figure 7:**
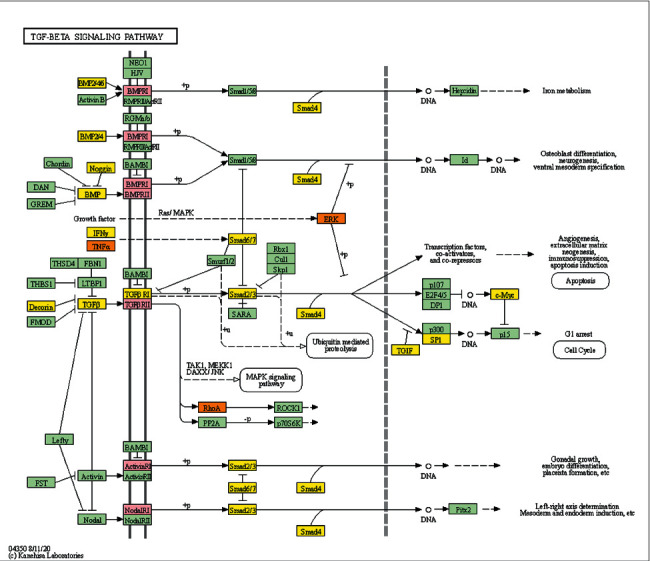
TGF-beta signaling pathway (modified from hsa04350. JWBGZP target is marked as pink; PMO gene is marked as yellow; JWBGZP-PMO target is marked as orange).

The JWBGZP potential target and PMO-related genes were input into DAVID for enrichment analysis (Figure 4). The biological processes include oxidation-reduction process, aging, response to hypoxia, response to ethanol, negative regulation of cell proliferation, negative regulation of apoptotic process, inflammatory response, steroid-hormone-mediated signaling pathway, response to estradiol, glucose metabolic process, glucose homeostasis, positive regulation of ERK1 and ERK2 cascade, one-carbon metabolic process, skeletal system development, etc. The cell components include extracellular exosome, extracellular space, cytosol, mitochondrion, extracellular region, plasma membrane, postsynaptic membrane, integral component of plasma membrane, GABA-A receptor complex, mitochondrial matrix, voltage-gated sodium channel complex, cell surface, chloride channel complex, neuronal cell body, etc. The molecular function include drug binding, extracellular ligand-gated ion channel activity, L-ascorbic acid binding, steroid hormone receptor activity, GABA-A receptor activity, receptor binding, ATP binding, oxidoreductase activity, RNA polymerase II transcription factor activity, ligand-activated sequence-specific DNA binding, cytokine activity, etc. The signaling pathway include neuroactive ligand-receptor interaction, osteoclast differentiation, cAMP signaling pathway, adipocytokine signaling pathway, metabolic pathways, FoxO signaling pathway, insulin resistance, hepatitis B, TNF signaling pathway, rheumatoid arthritis, HIF-1 signaling pathway, AMPK signaling pathway, insulin signaling pathway, and NF-kappa B signaling pathway (Figure 5). The signaling pathways are shown in Figure 6. The gene of TGF-beta is shown in Figure 7. The details are shown in Table S3.

Through PPI target network analysis, the important targets of JWBGZP for the treatment of PMO are obtained: MAPK1, AKT1, PIK3CA, PIK3R1, MAPK8, JAK2, etc. Among them, MAPK1 and MAKE8 are members of the MAPK signaling pathway, which can preclude osteoblast apoptosis through the mitochondrial pathway [27]. MAPK1, also known as ERK2, can mediate osteoblast differentiation through the ERK1/2 pathway [28, 29]. AKT1 is a member of the gene family of AKT (protein kinase B). Studies have found that after the AKT1 gene knockout treatment in rats, the rats have restricted bone development. It shows that AKTI may play a role in promoting bone growth in bone development [30], and at the same time, AKT1, PIK3CA, and PIK3R1 jointly act as members of the PI3K-Akt signaling pathway, and play a regulatory role in the process of bone metabolism through this pathway [31, 32]. JAK2 is a member of the JAK/STAT signal pathway, which can cause bone destruction by participating in the inflammatory response [33]. The TGF-*β*1/Smads signaling pathway is an important signaling pathway between bone resorption and bone formation in the process of bone metabolism, and it plays an important role in the balance regulation of bone metabolism [34]. The smad protein family is the only transduction molecule that conducts signals in TGF-*β*1 cells. Smads can combine with other transcription molecules to transfer the response signal from the cell membrane to the nucleus. Among them, Smad4 is a common molecule required for TGF-*β*1 signal transduction [35].

The results of GO enrichment analysis can infer that these key targets mainly play a role in the plasma membrane, membrane raft, and cell nucleus. JWBGZP may exert molecular functions such as kinase activity, protein binding, and protein kinase binding through positive regulation of nitric oxide biosynthesis, signal transduction, protein phosphorylation, and other biological processes. The PI3K-Akt signaling pathway is an important signaling pathway in the process of bone metabolism. It has the effects of mediating osteoblast proliferation [36, 37], osteoblast differentiation [38], and interfering with osteoclast apoptosis [39]. It is worth mentioning that the key targets AKT1, PIK3CA, and PIK3R1 selected in this study are all members of this pathway, and the research results are mutually verified. The HIF-1 signaling pathway has previously been proven to promote bone formation [40]. In animal experiments, it was found that the HIF-1 signaling pathway was inhibited, and the degree of osteoporosis in experimental mice was improved. While the osteoclast index of bone metabolism decreased, the index of bone metabolism osteogenesis also decreased [40]. The HIF-1 signaling pathway may be mainly involved in the regulation of osteoclast activity and exert its anti-PMO effect. This research also involves three hormonal signaling pathways. Among them, the estrogen signaling pathway promotes the proliferation and differentiation of osteoblasts by up-regulating the expression of OPG/RANKL through estrogen receptors [41], and at the same time, up-regulating CFTR to induce the apoptosis of osteoclasts, which has a two-way regulation effect on bone formation and osteoclast. The prolactin signaling pathway acts on the reproductive axis to inhibit the synthesis of steroid hormones and mediate bone metabolism [42]. Thyroxine directly or indirectly acts on chondrocytes, osteoclasts, and osteoblasts to achieve bone transformation [43].

In summary, the treatment of PMO by JWBGZP is to act on multiple gene targets through multiple active ingredients in JWBGZP. The functions of each gene are complex and diverse and regulate bone metabolism through different signaling pathways. This reflects the characteristics of multiple components, multiple targets, and multiple pathways in the treatment of PMO by JWBGZP and provides a reference for in-depth research on the mechanism of JWBGZP in the treatment of PMO in the future.

### 3.3. Serum E2 and Bone Density of Ovariectomized Female Rats

**Figure 8:**
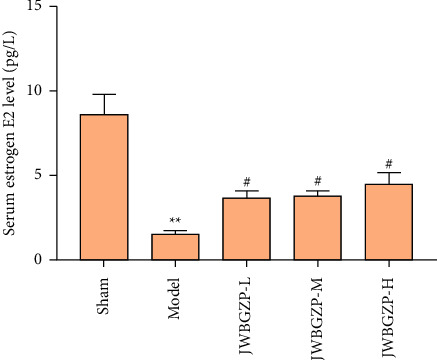
Serum E2 of ovariectomized female rats (compared with sham operation group, ^∗∗^*P* < 0.01; compared with model group, ^#^*P* < 0.05).

**Figure 9:**
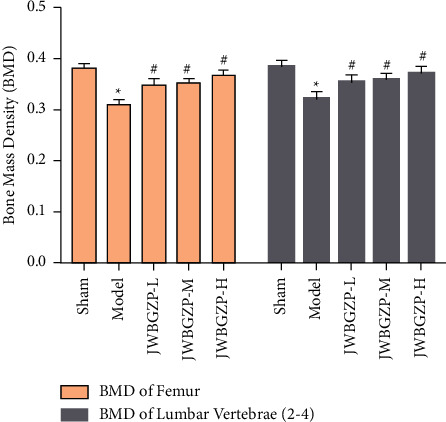
Bone density of ovariectomized female rats (compared with sham operation group, ^*∗*^*P* < 0.05; compared with model group, ^#^*P* < 0.05).

After castration, the serum E2 of the rats decreased significantly, which was statistically significant compared with the nonexperimental group (*P* < 0.01). After treatment with JWBGZP, E2 content increased significantly (*P* < 0.05) (Figure 8). Compared with the sham operation group, the femoral bone mineral density and lumbar bone mineral density of the model group were significantly lower (*P* < 0.05), indicating that the model was successful. Compared with the model group, the femoral bone mineral density and lumbar bone mineral density of the JWBGZP group increased (*P* < 0.05) (Figure 9).

### 3.4. Effect of JWBGZP on mRNA Expression of TGF-*β*1/Smad4 Pathway-Related Molecules

**Figure 10:**
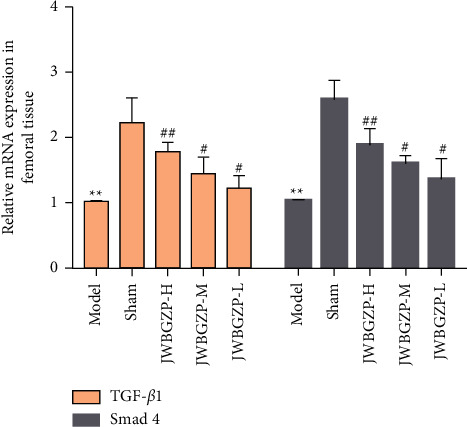
Effect of JWBGZP on mRNA expression of the TGF-*β*1/Smad4 pathway-related molecules (compared with sham operation group, ^∗∗^*P* < 0.01; compared with model group, ^#^*P* < 0.05, ^##^*P* < 0.01).

Compared with the sham operation group, the expression levels of TGF-*β*1 and Smad4 mRNA in the femoral tissue of the model group were significantly down-regulated (*P* < 0.05). Compared with the model group, the expression levels of TGF-*β*1 and Smad4 mRNA in the bone tissue of the JWBGZP group increased (*P* < 0.05; *P* < 0.01) (Figure 10).

### 3.5. Effect of JWBGZP on Protein Expression of TGF-*β*1/Smad4 Pathway-Related Molecules

**Figure 11:**
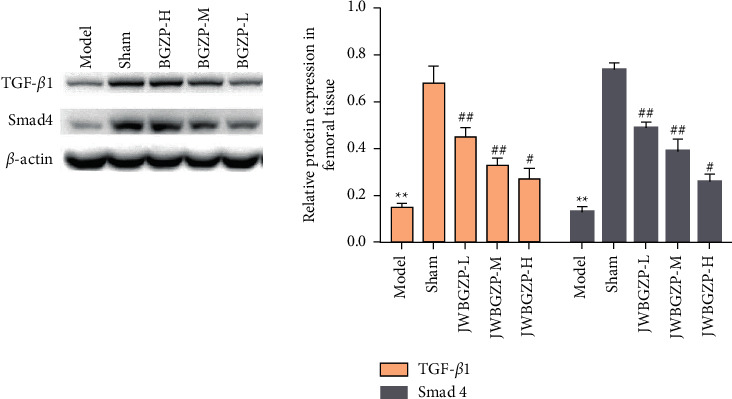
Effect of JWBGZP on mRNA expression of the TGF-*β*1/Smad4 pathway-related molecules (compared with sham operation group, ^∗∗^*P* < 0.01; compared with model group, ^#^*P* < 0.05, ^##^*P* < 0.01).

Compared with the sham operation group, the protein expression levels of TGF-*β*1 and Smad4 in the femoral tissue of the model group were significantly decreased (*P* < 0.05). Compared with the model group, the protein expression levels of TGF-*β*1 and Smad4 in the femoral tissue of JWBGZP increased (*P* < 0.05, *P* < 0.01) (Figure 11).

### 3.6. Osteoblast Identification

**Figure 12:**
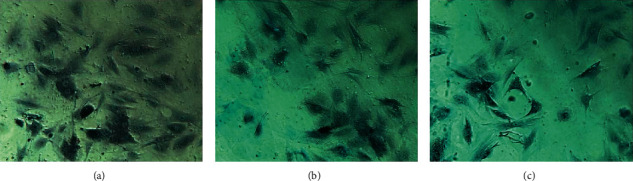
Alkaline phosphatase staining (NAP) (400X). (a–c) Different fields of view.

After osteoblasts were cultured for 6 days; alkaline phosphatase staining solution (azo coupling method) was used to stain osteoblasts to determine the alkaline phosphatase activity. The cytoplasm is stained blue, and the nucleus is stained green, indicating that the cell may be an osteoblast (Figure 12).

**Figure 13:**
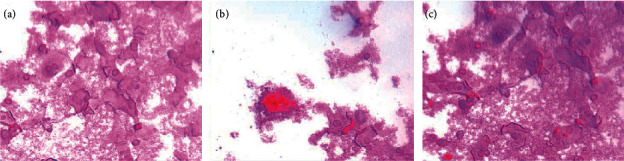
Alizarin red Staining (ARS) (400X). (a–c) Different fields of view.

Osteoblasts were cultured for 18 days and stained with alizarin red. The cell culture group with more calcified nodules was selected and stained with 0.1% alizarin red-tris-HCL at 37°C for 30 min. Alizarin red is a negative staining solution, which can combine with calcium salt deposits to form a large number of orange-red nodules with clear boundaries. The orange-red nodules and clear boundaries are used as the standard, and the differentiation and proliferation are counted under a 400x optical microscope. A large number of orange-red nodules can be seen in the picture, which further confirms that the cells are osteoblasts and have a large amount of calcium deposits (Figure 13).

### 3.7. Expression of TGF-*β*1 and Smad4 Protein Determined by Immunohistochemistry

**Figure 14:**
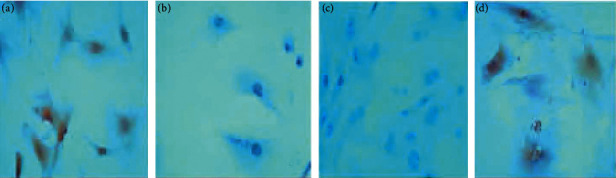
TGF-*β*1 expression (immunohistochemistry, 400X). (a) Blank control group; (b) sham operation group; (c) model group; (d) JWBGZP group.

**Figure 15:**
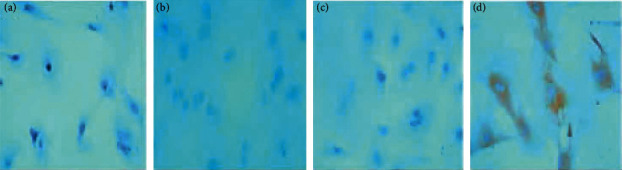
Smad4 expression (immunohistochemistry, 400X). (a) Blank control group; (b) sham operation group; (c) model group; (d) JWBGZP group.

**Figure 16:**
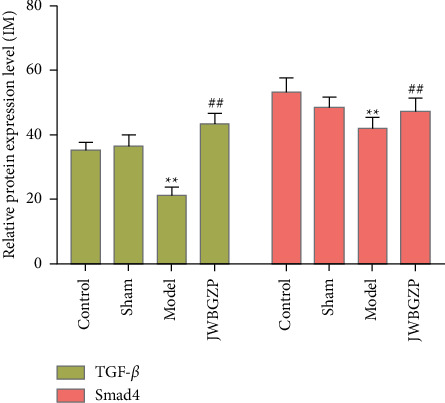
TGF-*β* and Smad4 expression (^∗∗^compared with sham operation group, *P* < 0.01; ^##^compared with model group, *P* < 0.01).

Immunohistochemistry was used to detect TGF-*β*1 protein and Smad4 protein in osteoblasts. The slides were randomly selected from 5 fields of view using a microscope camera system under a 400-fold light microscope for image analysis, and the contents of TGF-*β*1 protein and Smad protein in each field were detected, and the average was taken. Through data analysis, it can be seen that the expression levels of TGF-*β*1 and Smad4 proteins in the JWBGZP group were significantly higher than those in the blank control group and the sham operation group, while the expression levels in the model group were the lowest. This indicates that JWBGZP may increase the content of TGF-*β*1 protein and Smad protein in osteoblasts (Figures 14to 16).

The TGF-*β*-Smad signaling transduction pathway consists of the TGF-*β* signal molecule and the Smad protein. The TGF-*β* signal molecule conducts signal transduction through the transmembrane receptor complex [44]. There are mainly two types of TGF-*β* receptors (T*β*R), type I and type II, which are necessary for its conduction process [45], and its functions involve extracellular matrix deposition, growth inhibition, and apoptosis. The TGF-*β* gene exerts antiosteoporosis by regulating the function of bone deposition and osteoclasts, and the TGF-*β* protein expressed by it promotes the proliferation and differentiation of osteoblasts and promotes the synthesis of extracellular matrix to affect the formation of bone [46]. In addition, TGF-*β* also has an active bone repair function, and studies have found that it is highly correlated with the concentration of estrogen [47]. Smad signaling protein is a transcription factor that plays an important role in the intracellular signal transduction of members of the TGF-*β* superfamily. All TGF-*β* signaling molecules must be mediated by Smad4 protein to enter the nucleus, and Smad6 and 7 are inhibitors of this pathway [48, 49]. This study found that under the action of JWBGZP, the expression level of the TGF-*β*-Smad signal transduction pathway in osteoblasts was significantly higher than that of other groups. This proves that TGF-*β*-Smad is an important pathway for JWBGZP to treat postmenopausal osteoporosis, and JWBGZP can increase the expression level of this pathway.

This research explored the molecular mechanism of JWBGZP in the treatment of osteoporosis through network pharmacology and found that JWBGZP may improve the pathological process of osteoporosis through TGF-*β*-related signaling pathways through in vivo and in vitro experiments. However, this study still has shortcomings. For example, in this study, the exploration of the downstream signal pathway transduction of the TGF-*β* signal pathway is still not deep enough. In the future, we hope to design inhibitors and siRNAs targeting the TGF-*β* signaling pathway to further study in vivo and in vitro osteoporosis models.

## 4. Conclusion

JWBGZP may be involved in PI3K-Akt, HIF-1, estrogen, prolactin, and other signaling pathways, regulate MAPK1, AKT1, PIK3CA, JAK2, and other gene targets, regulate bone metabolism, and thereby treat PMO.

## Figures and Tables

**Table 1:** The primer sequences.

Gene	Sequence forward	Sequence reverse
TGF-*β*1	5′-CCAAGGAGACGGAATACAGG3′	5′-GTGTTGGTTGTAGAGGGCAAG-3′
Smad4	5′-TCAGCCAGCTACTTACCACCA-3′	5′-ACACGTCCCCTTCACCTTTAC-3′
*β*-Actin	5′-TGTATGCCTCTGGTCGTACCAC-3′	5′-ACAGAGTACTTGCGCTCAGGAG3′

## Data Availability

All datasets for this study are included in the manuscript and the supplementary files.

## Supplementary Materials

Supplementary Materials Table S1: JWBGZP potential components and targets. Table S2: PMO genes. Table S3: enrichment analysis of JWBGZP-PMO PPI network (*s*).
